# Health in the Skies: A Narrative Review of the Issues Faced by Commercial Airline Pilots

**DOI:** 10.7759/cureus.38000

**Published:** 2023-04-22

**Authors:** Piercarlo Minoretti, Enzo Emanuele

**Affiliations:** 1 Preventive Medicine, Studio Minoretti, Oggiono, ITA; 2 Pathology and Laboratory Medicine, 2E Science, Robbio, ITA

**Keywords:** occupational health hazards, review article, prevention, screening, airline pilots

## Abstract

While the health of airline pilots is crucial to ensuring the safe travel of millions of people worldwide, they remain vulnerable to a variety of health challenges due to the nature of their job. The purpose of this narrative review is to provide a comprehensive summary of the most common health issues experienced by commercial airline pilots. By examining the published literature on this topic, we sought to identify areas where further research is needed to understand better the health risks associated with being a pilot and to develop effective interventions to address these risks. We also highlight how recent technological advances in digital health can be leveraged to conduct research into the potential usefulness of telehealth assessments for identifying occupational hazards in the aviation sector and providing targeted interventions. Overall, addressing the challenges of taking care of pilots’ health and ensuring public safety will require a collaborative effort among airlines, governments, and regulators. Prioritizing pilot health and safety can actually improve profitability in the aviation sector by reducing costs associated with absenteeism, turnover, and accidents.

## Introduction and background

Commercial airline pilots are trained to fly various types of aircraft, including airplanes and helicopters, on short or long-haul flights [1,2]. They are responsible for transporting passengers and/or cargo safely and efficiently, while following strict safety procedures and regulations. A pilot’s job requires a high level of skill, training, and attention to detail, and involves numerous tasks and responsibilities beyond flying the aircraft [3,4]. Pilots must be able to assess and manage risks, communicate effectively with air traffic control, ensure passenger safety, and manage the flight crew.

In recent decades, the aviation industry has faced increasing competition and pressure to reduce costs, which has raised concerns about the working conditions and job security of pilots [5]. These concerns have potential implications for the safety and efficiency of flights. As airlines seek to reduce costs, they may implement cost-cutting measures (e.g., implementing contract work arrangements and applying pay-to-fly schemes) that can negatively impact pilots’ performance and the safety of flights [5]. In addition, these changes can create instability and uncertainty for pilots, which can negatively impact their job satisfaction and motivation. This can lead to increased turnover rates and difficulty in recruiting and retaining qualified pilots, which can further impact the safety and efficiency of the aviation industry. Given this scenario, the well-being of commercial airline pilots is paramount to ensuring the safety and stability of the aviation sector. This comprehensive narrative review strives to offer an extensive examination of prevalent health concerns that may be directly linked to the pilots' professional environment or exacerbated by their working conditions. Moreover, our objective is to identify key areas where targeted interventions can bolster pilots' overall health and well-being. Lastly, we delve into prospective avenues for further investigation within this crucial field.

## Review

Methodology

In order to review the available literature on the topic of airline pilots’ health, we searched electronic databases including PubMed, Web of Science, and Scopus for all studies published from January 1990 until July 2022 using a combination of the following terms: pilots, airlines, health, disease, prevention, and aircraft. A health information specialist (PM) performed the search with appropriate wildcards to take into account plurals and variations in spellings. The titles and abstracts of 605 articles were reviewed. The intent of reviewing the relevant literature on the subject of pilots’ health was not to conduct a systematic review. When a meta-analysis or a systematic review was already conducted on a specific topic (e.g., fatigue, melanoma, or cardiovascular diseases), we mainly referred to the conclusions reported in these articles, without resorting to a narrative summary of the included studies. This ensured that the review covered substantial relevant literature on the subject and was not confined to one research methodology. The sections that follow are not meant to be comprehensive but rather to emphasize the aspects that are most important in investigating effective strategies for safeguarding pilots' health and ensuring the safety of the aviation sector. An in-depth examination of work-related disabilities and the impact of the coronavirus disease 2019 (COVID-19) pandemic on the aviation industry was beyond the scope of the review.

Results

Circadian Disruption and Sleep Problems

Circadian desynchronization commonly experienced by pilots may interfere significantly with human well-being and homeostasis [1]. During long-haul flights, the disruption of the body's circadian rhythms, which regulate various physiological functions, leads to the phenomenon known as "jet lag". This condition is marked by symptoms such as drowsiness, insomnia, exhaustion, irritability, and diminished cognitive performance [2]. Work-related perturbation of the sleep/wake cycle may result in several sleep-related problems, including sleepiness, impaired sleep quality, insomnia, fatigue, and obstructive sleep apnea (OSA). Growing evidence indicates that persistent sleep disorders are highly prevalent among pilots. On analyzing a sample of 435 Portuguese pilots, Reis et al. [3] found that the prevalence of sleep complaints and daytime sleepiness was 34.9% and 59.3%, respectively. In a study conducted by Alzehairi et al. [4] on 344 pilots in Saudi Arabia, approximately half of the sample was at risk for insomnia. They also found that older and more experienced pilots were less likely to report impaired sleep quality and sleepiness [4]. Another large study by Pellegrino et al. [5] investigating 1234 Brazilian commercial aviation pilots reported a 48.2% prevalence of poor sleep quality. The main predictors of impaired sleep quality included moderate and great need for recovery after work, high frequency of technical delays, being insufficiently physically active and sleeping 6−8 hours and < 6 hours on days off, over five consecutive night shifts, and difficulty commuting to work [5]. These results were broadly confirmed by Sallinen et al. [6], who identified flight duty period timing and inadequate sleep as the main self-reported reasons for on-duty sleepiness among short- and long-haul pilots. OSA is another sleep-related condition that can have a negative impact on pilots' job performance as it involves recurring instances of apnea and hypopnea while sleeping [7]. In a study by Alhejaili et al. [8], OSA was identified by home sleep testing in 69% of Saudi-based airline pilots. On analyzing 103 pilots after a long-haul night-time flight using daytime polysomnography, Han et al. [9] found that 73 (70.9%) had moderate-to-severe daytime OSA despite no known previous history. Elevated body mass index and cumulative flight time were identified as independent predictors [9]. Pilots should be educated about the long-term importance for health of obtaining adequate sleep on off-duty days. Regular assessment by occupational physicians with robust and objective tools (apnea-hypopnea index, apnoeas and hypopnoeas/time in bed, oxygen desaturation index) should be implemented to detect and treat symptoms of insomnia or excessive sleepiness. Future research is also needed to investigate whether specific pharmacological and/or non-pharmacological sleep health interventions can have a positive effect on alertness and reduce on-duty sleepiness.

Fatigue

Fatigue, a complex subjective phenomenon reflecting a high physical and mental load, is commonly reported by airline pilots, either alone or in combination with sleep-related issues [10]. In recent years, the number of fatigue related safety incidents has grown, with personnel sighting fatigue and sleep loss as the causation of operational errors such as landing on incorrect runways or fuel miscalculations [11]. This may be further exacerbated by high workloads and unfavorable environmental conditions. On examining the prevalence and risk factors for fatigue in a large sample of 502 pilots, van Drongelen et al. [12] reported that 29.5% met the criteria for being fatigued. The main risk factors included older age, moderate alcohol consumption, limited physical activity, poor work-life balance, more need for recovery, and a lower perceived health [12]. In a study that involved Portuguese airline pilots, Reis et al. [13] found that medium/short-haul pilots presented the highest levels of total and mental fatigue; this may possibly be explained by the fact that medium/short-haul pilots perform more take-offs and landings per duty period than long-haul pilots, resulting in a higher workload in the former group.

Psychological Distress and Psychiatric Conditions

Fatigue and poor work-life balance are common sources of psychological distress in airline pilots. Persistent disruption of circadian rhythms and stressful working conditions may also lead to clinically significant mood disorders, including major depression [14]. In recent years, this has been further exacerbated by changes in job stability during the COVID-19 pandemic. By using the 12-item general health questionnaire (GHQ-12), Alaminos-Torres et al. [15] investigated psychological distress in Spanish airline pilots amid the COVID-19 aviation crisis. The mean total score on the GHQ-12 in the entire study sample (342 responders) was close to established cut-off point for clinically significant psychological distress. In addition, the presence of psychological distress was associated with a less favorable employment status (i.e., being unemployed or furloughed). Severe psychiatric conditions may also increase the probability of dangerous behaviors during piloting [15]. The 2015 Germanwings Flight 9525 disaster, in which the 27-year-old copilot may have locked the captain out of the cockpit and deliberately crashed the plane [16], has dramatically highlighted the sensitive subject of pilots’ mental health, especially with respect to major depression and suicidal thoughts. In a review of 20 studies conducted after the Germanwings Flight 9525 crash, the prevalence of depression in commercial airline pilots was found to range between 1.9% and 12.6% [17]. Factors that negatively impact the mental health of pilots include substance abuse, experiencing verbal or sexual abuse, fatigue, and disruption in sleep circadian rhythms. Since fear of negative career impacts may prevent flying pilots from seeking help for high levels of psychological distress or psychiatric conditions, it is recommended that civil aviation authorities take measures to raise awareness and provide assistance for mental health treatment aimed at prevention [17].

Lower Back Pain

Physical factors in the cockpit, including prolonged seating postures, lifting, and whole-body vibration, impose postural strains on lumbar spines and can result in lower back pain (LBP) [18]. Albermann et al. [19] examined the prevalence of LBP in 698 German airline pilots using an anonymous online survey. The prevalence of chronic LBP was as high as 82.7%, with 51% reportedly having current LBP at the time of analysis. In addition, the prevalence rates of acute and subacute LBP were 8.2% and 2.4%, respectively. The main risk factor for acute nonspecific LBP was a total time spent flying greater than 600 h within the previous year, although causation was not firmly established. The presence of LBP was associated with more disability and lower functioning [19].

Venous Thromboembolism

Prolonged seated immobility during long-haul flights is a well-known risk factor for venous thromboembolism (VTE) [20]. While this issue has received extensive attention among passengers, the published literature on commercial airline pilots remains limited. However, the effectiveness of prophylaxis critically depends on awareness of potentially life-threatening condition. Kilic and Soran [21] evaluated actionable awareness regarding the risk of VTE among a large cohort of 427 airline pilots using a dedicated questionnaire. Surprisingly, 63.9% of the study participants were unaware of flight-associated VTE, thereby leading to missing the window of opportunity for prevention. Pilots aged between 20 and 40 years were much less aware of VTE compared with pilots aged ≥ 41 years and may therefore be more vulnerable [21]. Improvement in prevention strategies for VTE may be achieved by providing expanded training and focusing on younger pilots in this field.

Malignant Melanoma

Malignant melanoma (MM) of the skin is thought to arise from different causal pathways and the potential causative role of cosmic and ultraviolet (UV) radiation has not been entirely elucidated [22]. Occupational radiation exposure in airline pilots and cabin crew have been repeatedly investigated in relation to the risk of MM. In general, exposure to UV in the cockpit is mainly related to flight duration and the presence of direct sunlight [22]. In terms of effective dose, the yearly radiation doses for airline crew from commercial flights are estimated to be less than 4-5 mSv, with a range between 0.2 and 5 mSv [23]. In a meta-analysis by Sanlorenzo et al. [24] conducted on 266431 participants, pilots and cabin crew were found to have approximately twice the incidence of MM compared with the general population. These results were largely confirmed by the recent systematic review and meta-analysis by Miura et al. [25], who showed that airline pilots and cabin crew have about twice the risk of MM and other non-melanoma skin cancers than the general population, with pilots more likely to die from MM. A disproportionately elevated mortality from MM compared with the general population has also been reported in a cohort of USA commercial airline cockpit crew [23]. However, most of the evidence was collected several years ago and their relevance to contemporary levels of MM risk is uncertain. Future studies on the risk of developing MM in pilots should dissect the role of UV attenuation of aircraft windshields, cumulative flight time, and destinations [26]. Additionally, leisure and recreational outdoor time should be taken into account as these factors may act as potential confounders. In the future, setting the acceptable levels for occupational exposure of pilots to cosmic and UV radiation will require the identification of a reliable biomarker. Using a sample of 83 male airline pilots, Grajewski et al. [27] estimated median cumulative absorbed dose and the occurrence of chromosome translocations as a biological marker of cumulative exposure to ionizing radiation. They found a positive association between translocation frequency and the absorbed dose, which was limited to commercial flying [27].

Cardiovascular Diseases

Airline pilots experience occupational exposures that are widely recognized as having strong connections to cardiovascular disease (CVD). In a recent systematic review of 48 studies conducted in 20 different countries, Wilson et al. [28] analyzed the distribution of physiological, behavioral, and psychological cardiometabolic health risk factors in a pooled sample of 36958 pilots. They found that, compared with the general population, airline pilots had high prevalence of overweight and obesity, metabolic syndrome, type 2 diabetes, insufficient physical activity, elevated psychological fatigue, insufficient fruit and vegetable intake, and regular alcohol consumption [28]. Addressing these highly prevalent risk factors for CVD may reduce occupational morbidity, potentially reducing medical conditions causing medical incapacity and loss of license. Given the risk profile evidence of the airline pilot population, research has recently focused on biochemical markers of CVD. Increased levels of C-reactive protein and atherogenic lipids, including low-density lipoprotein cholesterol, have been reported in pilots [29]. However, the question as to whether job strain and chronic stress can have significant and direct influence on inflammatory markers and cholesterol levels remains unanswered. Efforts to alleviate the impact of cardiovascular disease (CVD) among pilots are crucial for the sustained well-being of the aviation sector. Consequently, regulatory bodies should strive to bridge any gaps between their recommendations for pilots and those for the general population with elevated cardiovascular risk, ensuring optimal preventive measures and support.

Tinnitus

Tinnitus, a symptom characterized by an auditory perception unrelated with any physical source, is commonly reported by subjects with prolonged exposure to occupational noise [30]. Airline pilots with tinnitus may suffer several degrees of distress, being a source of distraction and interfering with communications in the cockpit and with air traffic control. In a study conducted among 418 male and 42 female pilots on duty in a Swedish airline, Lindgren et al. [31] showed that 40% of the respondents had experienced tinnitus for more than five minutes during the previous year. They also found that 18% had constant or severe tinnitus, whereas 12% had at some time visited a doctor for tinnitus-related problems. As expected, pilots with tinnitus were more likely to report themselves disturbed by noise in the cockpit. The main predictors of tinnitus included age, impulse noise, and hearing impairment at 3, 4, and 6 kHz. However, no association with aircraft type or work as a military pilot was observed [31].

Gastrointestinal Disorders

Meal times are important synchronizers of the human life and disruptions in circadian rhythms coupled with arduous working environment have been associated with gastrointestinal disorders (GIs) in airline pilots. In a USA study conducted from 2001 through 2013 in the population of active duty Air Force pilots, esophageal disease and dyspeptic conditions were the two most frequently encountered GIs [32]. In an investigation involving 354 pilots of a Swedish airline company, 9.9% reported poor appetite, 15.2% heartburn, 12.4% diarrhea, 62.1% bloating, 9.3% constipation, and 14.4% epigastralgia over a three-month period [33]. The main predictors of GI complaints included in-somnia, BMI, smoking, female sex, and milk consumption [33]. On analyzing 212 male pilots working for a Chinese large civil airline company, Li et al. [34] reported a 39.22% prevalence for functional GIs. In multivariable analysis, the flight level, high-salt food pattern, and sleep performance were identified as independent predictors [34].

Other Health Issues

Owing to their exposure to increased levels of cosmic radiation [35], it has been hypothesized that airline pilots and flight attendants could be at higher risk of developing thyroid [36] or breast [35] malignancies. However, increased rates of breast cancer among flight attendants have been attributed to reproductive factors such as nulliparity [35]. Additionally, a recent meta-analysis [36] showed that airline crews do not have a significantly elevated risk of thyroid cancer incidence or mortality relative to the general population. In a study conducted on pilots in one Swedish commercial airline [37], work-related psychosocial risk factors have been related to a variety of musculoskeletal symptoms. In addition, pilots and airline crews can be exposed to a wide range of potential chemical irritants and airborne pollutants, which may lead to dermatitis [38]. Finally, increased rates of cataract have been reported among pilots [39]. Even without a decrease in visual acuity, mild or early lens opacities can cause significant glare and haze and alter color vision, potentially compromising pilot performance [40]. A recent Australian study showed that cataracts in pilots ≥ 60 years was generally bilateral and of mild severity, whereas cataracts in pilots aged less than 60 years were more likely to be unilateral and of greater severity [41].

Health-Related Quality of Life

Health-related quality of life (HRQOL) is a multi-dimensional indicator of overall health that captures information on a person’s physical and mental health status, as well as on the impact of health status on quality of life [42]. The main advantage of HRQOL is that it expands upon traditional objective clinical measurements to provide subjective experiences and perceptions of health encompassing the physical, mental, and social dimensions [42]. There are limited published data on HRQOL in commercial airline pilots. Liu et al. [43] investigated HRQOL and its related factors in a sample of 373 pilots recruited from a Chinese commercial airline. Physical activity as well as fruit and vegetable intake were positively associated with HRQOL, whereas time-zone flights, smoking, alcohol drinking, and dyslipidemia showed a negative association. The overall perception of HRQOL in Chinese commercial airline pilots is therefore determined by individual-level lifestyle factors (i.e., physical activity, fruit and vegetable intake, smoking, and alcohol drinking), a health-related factor (i.e., dyslipidemia), and a work-related factor (time-zone flights) [43]. Albeit preliminary, these findings could serve as an initial guide for employment policymakers to implement measures that ensure a suitable HRQOL for airline pilots.

Discussion

Based on the published literature, the occupational milieu experienced by commercial airline pilots appears to be linked with several potential health issues which are only partially addressed by current interventions (e.g., workplace peer support programs). Another major issue is that preventive surveillance programs in the aviation industry may differ across countries and different civil aviation authorities. In light of this situation, it is imperative to conduct further research to examine the discrepancies and establish a unified understanding comprehensively. Enhanced collaboration among various authorities is also essential to ensure effective decision-making and alignment of policies. In general, effective prevention strategies against the full range of hazards encountered by pilots during their job should be aimed at reducing risk factors and strengthening protective measures. Apart from the specific paths for preventative interventions outlined below, the paramount contribution of guidelines released from civil aviation authorities for protecting pilots’ health should be adequately emphasized. Ideally, pragmatic action to improve pilots’ working conditions that cause common sleep- and fatigue-related health problems, including avoidance of irregular work schedules, optimization of work organization, and guarantee of adequate sleep. In light of recent technical advances, incorporating remote telehealth assessments and monitoring should also be explored in future studies as a meaningful strategy to address the gap in health care access that is still common for airline pilots [44], especially on long-haul routes.

Reducing the Risk of Sleep and Mental Health-Related Problems

Occupational physicians should inform airline pilots about proper sleep habits as well as on the relationships between sleep disorders, fatigue, and depression [4-11]. Current health surveillance activities should also include adequate screening programs for sleep disorders and OSA. Physicians should also promote awareness of mental health-related problems to increase understanding and reduce stigma [45]. Unfortunately, discussions of severe depressive symptoms may be off-putting for pilots who can fear serious negative effects on their employment status or even license revocation. In order to promote mental health and wellbeing among pilots, it is important to provide them with a range of resources and support services. This may include access to counseling and therapy, as well as other forms of support such as peer-to-peer support networks, stress management programs, and educational resources on mental health. Additionally, efforts should be made to reduce the stigma around seeking mental health treatment and to encourage pilots to prioritize their mental health and seek help when needed.

Reducing the Risk of Radiation-Induced Damage

The issue of radiation-induced damage in pilots is mainly related to the risk of developing MM. Airlines should follow the International Commission on Radiological Protection (ICRP) guidance on radiological protection from cosmic radiations in aviation to reduce occupational exposure of the cockpit crew [46]. Unfortunately, there is a little room to manoeuvre in terms of potential protective actions. Route selection by acting on altitude and latitude is frequently unfeasible due to technical and organizational considerations and should take into account both air traffic and weather conditions. Therefore, the ICRP recommends controlling exposures by adjusting the flight schedules of the most exposed individuals by consideration of flight time and route selection [46]. While annual and cumulative individual doses should be recorded, specific additional medical examinations are not currently recommended. The potential usefulness of sunscreens and oral chemopreventive agents (e.g., nicotinamide) for reducing the risk of MM in pilots warrants further investigation [47]. As part of general prevention efforts, campaigns and interventions for UV protection should also be focused on leisure time and corresponding activities.

Reducing the Burden of Cardiovascular and Metabolic Diseases

Aviation medical examiners , who typically conduct medical evaluations for airline pilots, should consider not only lifestyle risk factors but also work-related conditions associated with CVD, such as OSA and depression. By doing so, they can identify pilots who would greatly benefit from targeted prevention strategies. Traditional modifiable risk factors may be targeted by encouraging physical activity during leisure time, promoting healthy dietary choices, favoring smoking cessation, and preventing excess alcohol drinking. Due to the impaired cardiometabolic risk profile of airline pilots [28], future considerations should emphasize primary prevention of acute cardiovascular events, particularly in asymptomatic high-risk individuals. Development of risk scores that consider the widespread use of high-sensitivity troponin assays could allow medical examiners to estimate risk better [48]. When considered in combination with targeting of job-related conditions, these prevention strategies have the potential to provide a comprehensive framework for reducing the burden of CVD in pilots.

Strenghts and limitations

The current manuscript is an example of a narrative review with known limitations and caveats [49]. While systematic reviews are of higher quality, narrative reviews continue to be highly appreciated by the medical community [50] and leading journals still publish more narrative than systematic reviews [51]. In addition, an analysis of the effects of the COVID-19 pandemic on airline pilots was outside the scope of the current manuscript since this topic has been already covered in recent studies [52,53]. Despite these limitations, our review also has some notable strengths compared with a similar previous paper [54]. First, Kim and Lee [54] were chiefly focused on the general hazards of frequent flying, i.e., reduced atmospheric pressure, reduced available oxygen, noise, and vibration, and how they are addressed in the artificial environment within the aircraft. In our work, we adopted an entirely different perspective, i.e., a stance connected to occupational medicine and solely centered on airline pilots. Second, we provided a brief yet current summary of the state of airline pilots' health, more than a decade after Kim and Lee's publication [54]. This update was necessary and timely, given the ongoing changes and developments in the aviation industry and the potential impact on pilots' health and wellbeing. Finally, these authors provided a simple tabular list of the potential diseases faced by airline pilots, stewards, or cabin attendants [54], without extensive coverage of sleep problems, tinnitus, venous thromboembolism, or poor HRQOL, all of which were included in our paper.

## Conclusions

Emphasizing pilot health and safety can enhance profitability within the aviation industry by minimizing expenses related to absenteeism, employee turnover, and accidents. Nevertheless, the examined evidence reveals that pilots continue to face numerous health-related obstacles. Consequently, it is essential for airline pilots to focus on their well-being and proactively address these potential concerns. Airlines can proactively enhance the health and safety of their pilots by offering access to mental health services, establishing fatigue risk management systems, and encouraging healthy lifestyle choices. Future high-quality intervention studies will play a crucial role in determining effective strategies for boosting pilot well-being, ultimately leading to improved flight safety and performance. Leveraging recent technological advancements in digital health can empower the aviation industry to identify and mitigate occupational hazards. For instance, telehealth assessments offer a valuable tool for screening pilots for sleep disorders, which may contribute to fatigue and compromised performance. In addition, remote monitoring devices, such as wearable sensors, can track pilots' radiation or noise exposure, allowing for timely interventions if required. This holistic approach to health and safety in the aviation sector will ensure that pilots remain in peak condition, resulting in safer skies for all.
